# Distributed Steganography in PDF Files—Secrets Hidden in Modified Pages

**DOI:** 10.3390/e22060600

**Published:** 2020-05-28

**Authors:** Katarzyna Koptyra, Marek R. Ogiela

**Affiliations:** AGH University of Science and Technology, 30 Mickiewicza Ave., 30-059 Krakow, Poland; kkoptyra@agh.edu.pl

**Keywords:** steganography, pdf, secret sharing, distributed

## Abstract

This paper shows how to diffuse a message and hide it in multiple PDF files. Presented method uses dereferenced objects and secret splitting or sharing algorithms. It is applicable to various types of PDF files, including text documents, presentations, scanned images etc. Because hiding process is based on structure manipulation, the solution may be easily combined with content-dependent steganographic techniques. The hidden pages are not visible in typical application usage, which was tested with seven different programs.

## 1. Introduction

PDF is one of the most common used document formats nowadays. The main advantages of these files are preserving the appearance when the document is printed and using hyperlinks. On the other hand, PDF is not customizable and has many problems with changing a style, like increasing font size or adjusting contrast. Nevertheless, its popularity is growing together with the number of compatible software, including readers embedded in web browsers.

Not everyone knows that PDF files may hold secrets, too. Concealing data in an inconspicuous way is called *steganography* [1] and is mainly used to communicate secretly. This attribute differentiates it from cryptography [2,3]—when information is encrypted, it cannot be read, but everyone knows of its existence. In steganography, the primary goal is undetectability of secret message. This is realized with an ordinary file called carrier or container in which the message is embedded. Virtually any file may play this role, for example an image, video or HTML page [4].

**Figure 1:**
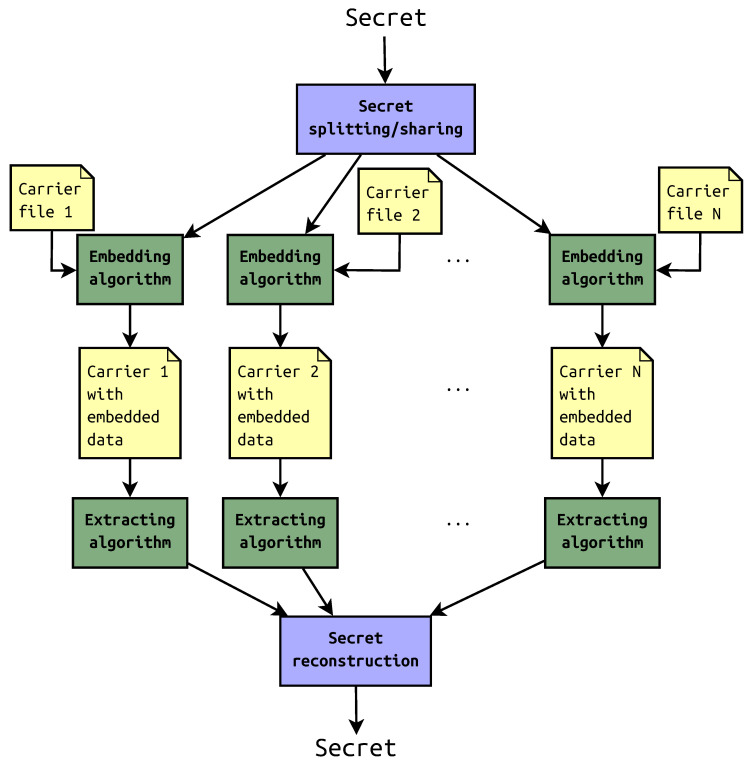
Distributed steganography scheme.

Sometimes, the steganography is used solely; in other cases, it is combined with other means of information security [5]. The one example is to disperse the message in the container with steganographic key. Another is distributed steganography in which the secret is split into some shares that are later embedded in multiple carrier files. The files with payload may be saved in remote locations or in a cloud. To reveal the secret, required number of shares should be recovered first. The general scheme of this approach is presented in Figure 1.

A few examples of steganographic algorithms designed to work on PDF files include: white text on white background, object overlapping or image steganography in existing pictures. These specialized solutions may be sufficient in some situations, but are not appropriate for many documents commonly found in real life applications, like pure text or scans. Presented steganographic method is more universal and may be used in any type of PDF file.

This paper describes how to split a payload and then conceal the shares in PDF files. Section 2 presents technical details of PDF format. Section 3 demonstrates general method of hiding pages in PDF documents with comprehensible example. Specific algorithms of secret splitting are shown in Section 4. Details of extracting the data from documents may be found in Section 5. The last section summarizes the entire article.

## 2. PDF Format

**Figure 2:**
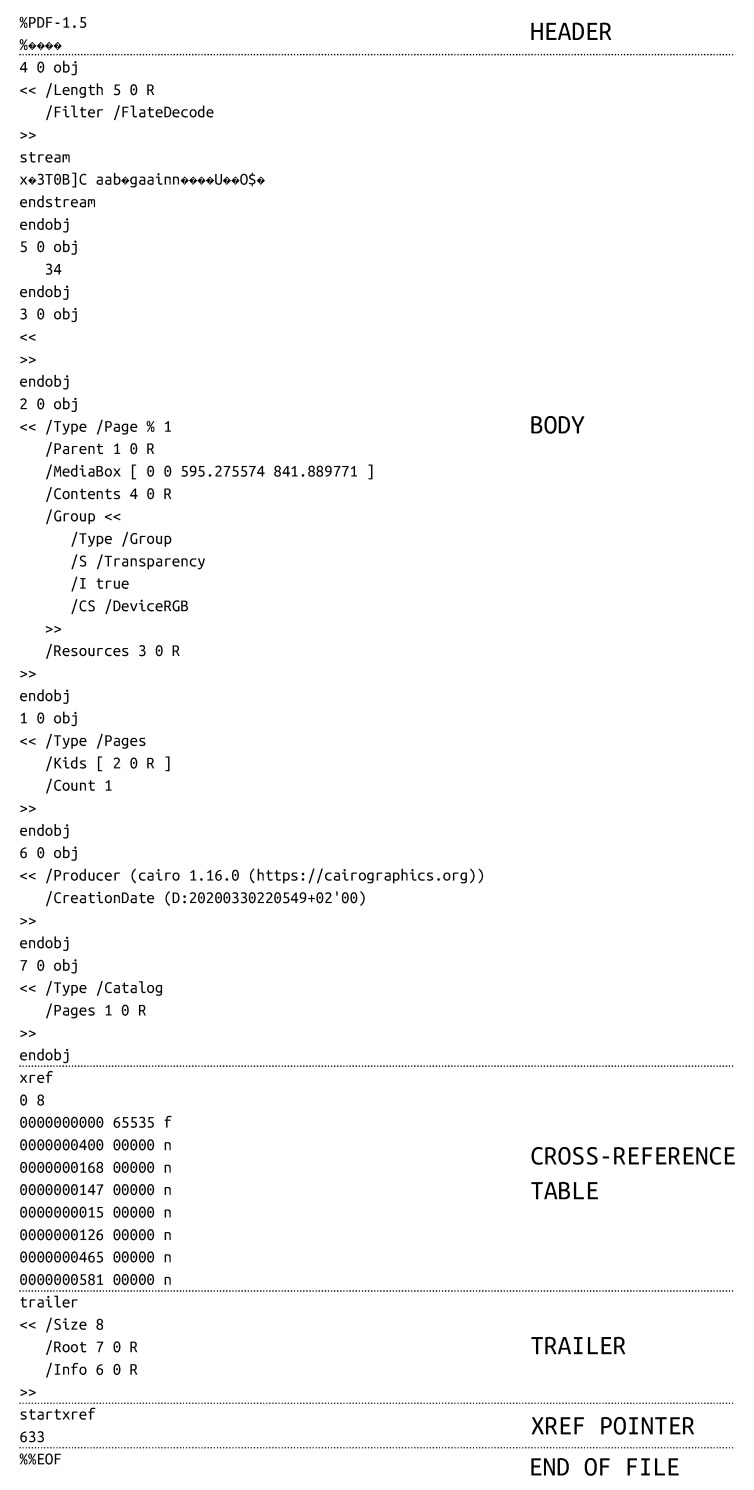
An example of simple PDF file and its parts. The lines on the scheme do not belong to the file; they were added for better readability.

PDF (Portable Document Format) is a popular document format that became open standard in 2008 [6]. It is text-based, but may (and usually does) contain binary streams. PDFs are consisted of a few parts: header, body, cross-reference table, trailer, xref pointer and end of file marker [7], as presented in Figure 2.

The header of PDF starts with a signature—%PDF- followed by version. It may be PDF-1.5 as in presented example, but older or newer version are possible as well. Another information that is optional in header is charset identifier. This is used by software to determine if the file is encoded in ASCII or not. When the identifier is present, it appears in second line of the file as 4 non-ASCII characters in a comment. (The comments in PDF are created with %, so the header itself is a comment!)

The next and most important part of PDF file is body section. Here are stored all contents normally visible in PDF reader. The body is consisted of *objects* which come in many types: boolean values, numbers, strings, arrays, dictionaries, binary streams and others. They are built in the following manner:


<number> <generation> obj
<content>
endobj


Each object has its own unique identifier (number). Generation of object is usually equal to 0 (unless the file has multiple revisions) and content very often lies between << and >> which is dictionary format.

Crossref (xref) plays a role of table of contents of the file. Its initial line stores two values: offset and number of objects (in our example from Figure 2 it is 0 8). After that there is a table in which each row corresponds to a single object and includes its offset and generation. The table has fixed length of 20 characters so that is easy to parse. The important information to note is that the first object is always null.

The objects form tree structure, so the order of elements in xref table does not necessarily correspond to their appearance in the document. Obviously, the initial element needs to be stored somehow to inform the parser where the content starts. This is what trailer is used for—it defines the reference to root object together with its size (count of indirect objects). When the trailer is parsed, it gives the location of root object which then allows to subsequently parse linked objects called kids.

The second to last element of PDF is xref pointer that indicates where xref table is located. It occupies two lines: first with startxref keyword and second with single number denoting the offset to xref.

PDFs end with %%EOF which is end of file marker. The important but easy to miss fact is that the percent character in this line is doubled.

## 3. Hidden Pages

The whole page(s) of PDF document may be hidden by modifying the tree structure of objects. The one idea is to remove reference to a certain page from its parent and to decrease the counter of pages. The content of the object remains untouched; only the reference is deleted. Let us consider the following example:


1 0 obj
<<
/Kids [3 0 R 11 0 R]
/Type /Pages
/Count 2
>>
endobj
		

This is a fragment from 2-pages PDF created to demonstrate concealing process. Presented object has two references to kids (id 3 and id 11) and the page counter is set to 2. To delete the second page, we need to remove the reference 11 0 R and additionally decrease page counter by 1. The resulting file is smaller than the original by 7 bytes (which is about 0.07% of total size). Here is how the object looks like after such modifications.


1 0 obj
<<
/Kids [3 0 R]
/Type /Pages
/Count 1
>>
endobj 


The new document was tested to see if it opens correctly and the number of pages was as expected. The tests included PDF readers that parse and show documents directly and also graphical software with ability to import PDF as raster or vector image. They were: Evince, Adobe Acrobat X, Okular, Firefox (web browser that may internally render PDFs)—group 1; GIMP, Inkscape and Draw from LibreOffice—group 2.

It turned out that every program was able to open the file and show total page count equal to 1 as it should. The only incident occurred during closing the document in Acrobat X. The application ask if it should save changes. But of course no changes were made, the file was just opened. Why this happened? Probably Acrobat X assumed that orphaned object was added to document in current session. If so, why a number of other applications processed this file normally and without any warnings? This is because most readers are written to be tolerant to various weirdness that sometimes appear in PDF files. There are even programs that accept malformed documents, for example with some elements missing. In this study Acrobat X was found the most strict software.

Another approach is applicable when pages are removed from the end of the file. This method is even simpler than previous one, as all we need to do is to decrease the page counter. Then the object would look like:


1 0 obj
<<
/Kids [3 0 R 11 0 R]
/Type /Pages
/Count 1
>>
endobj
 

In this case the reference was not cleared, so only 1 byte was modified and the file size did not change at all.

The tests from previous version were repeated on newly created document. Again, the document opened correctly in each program and the number of pages was equal to 1. This time Acrobat X did not show any dialog (as well as remaining applications) and no warnings were found. Lastly, new PDF was checked with command line program pdftk that was asked to extract the second page from the document. It raised the error that the input file has only 1 page—which confirmed correctness of the solution.

To sum up this section, both methods produce PDF documents with correct number of pages. Second technique (without clearing reference) was found more silent. The size of resulting file is identical or almost identical as the source PDF. Also, described algorithm of secret hiding works very efficiently in terms of complexity. No matter how many pages are concealed, counter modification is done in constant time.

### 3.1. Example of Adding and Hiding a Page in PDF

Presented technique is particularly useful when a new page (to be hidden) is added to preexisting PDF document. It requires two files: one carrier and one PDF with secret content. First the files are combined and then new pages are concealed as described earlier.

A comprehensible example demonstrate the whole process step by step. At the beginning, two files were prepared—simple 2-pages “Hello World” document that served as a carrier and secret document with text next to a picture.

Next, the documents were joined with pdftk (pdftoolkit):


pdftk ex1.pdf ex2.pdf cat output ex3.pdf
 

where ex1.pdf and ex2.pdf are input files and ex3.pdf is final document. Other methods of combining are also possible, such as pdfjam or various PDF printers. The resulting file had 3 pages, two from the container and one from secret document.

**Figure 3:**
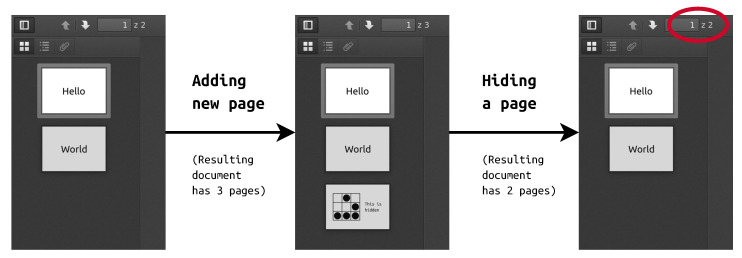
Example of adding and hiding secret page in PDF document.

Later secret content was hidden with decreasing total page counter. Indeed, the final document has only 2 pages, as presented in Figure 3. The screenshots show files opened in Firefox, but tests conducted with different software gave the same results.

The intermediate file was somewhat smaller than sum of carrier and secret file. This is because joining is not simple concatenation, but introduction of new objects and modification of crossref table. The final document had exactly the same length as the intermediate one, which was expected regarding hiding process as byte substitution.

**Figure 4:**
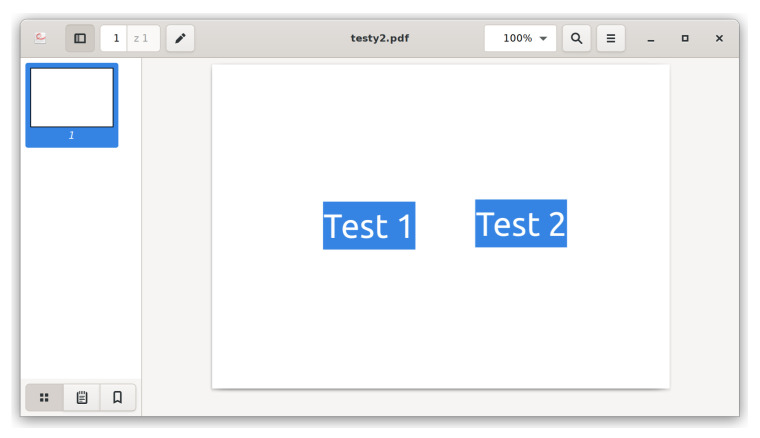
Example of different technique—hiding text by changing color or covering it with a rectangle.

Presented technique does not leave visual traces that user may accidentally bump into during normal application usage. This differentiate it from other known techniques, as setting text color identical to background or hiding the text behind another object. Figure 4 presents what happens when the hidden text is selected, either manually or with Ctrl+A. This is common practice if the user wants to copy an excerpt or when there is insufficient contrast and the content is hard to read. Normally both texts are invisible (one on the left is white, one on the right is covered with white rectangle), but with selection they are easy to spot. Such situation does not occur in described method which is based on structure manipulation.

## 4. Secret Sharing

Secret sharing techniques allow to divide a secret into several parts called shares that can later be joined to recover initial information [8]. Usually a certain number of shares is required to successfully reconstruct the secret [9]. This value is called threshold and it may be lower than total number of shares. In such case secret message may be reconstructed even when some parts are missing. It is also possible to create more complex solutions, including hierarchical [10], expandable [11,12] or multistage systems [13]. A majority of schemes use numeric secrets, but there are exceptions, for example matrices [14,15] or images [16].

The most known secret sharing algorithm is based on XOR operation. It provides perfect secrecy (any number of shares below the threshold is unable to find anything about the secret), but all shares are required to reconstruct secret message. For two parts, the first share is random binary data of length equal to secret length; the second share is a result of bitwise XOR operation on random data and the secret. The reconstruction is simply XOR of both shares.

XOR secret sharing is easy to demonstrate with text data that may be represented in ASCII, Unicode or other encoding standard. For example, converting “Hello” to ASCII codes gives 72, 101, 108, 108, 111. These numbers take part in share generation. Let us assume that we want three shares, so we need to create two sets of random numbers (of length equal to input text length, in this case 5). The values should be limited to range 0–255 as chosen encoding requires. The random sets are 20, 155, 255, 35, 218 and 38, 106, 229, 76, 174—they serve as share1 and share2. The last share is created as XOR of ASCII codes of “Hello” and two sets of random values, which is equal to 122, 148, 118, 3, 27. Now, to reconstruct initial text, one need to compute elementwise XOR of all three shares and transform the codes back to characters. This process is not presented here, but everyone is invited to test correctness of the algorithm with own hands.

In XOR method every share is necessary to recover the secret, which may be seen as disadvantage when some shares are missing or destroyed. In such cases schemes with threshold are the best solution. They have two parameters: *n*—total number of shares and *k*—number of shares sufficient to reconstruct the secret. An example of threshold system is encoding the secret in a polynomial. With *n* = 4 and *k* = 3, we need to create a polynomial of degree 2 and evaluate it at 4 chosen values. For instance, the secret is equal to 10 and our polynomial is $p\left(x\right)=-4x^{2}+2x+10$ (the secret is free term, remaining coefficients are random numbers). We choose 4 points that lie on polynomial: (1,8), (2,−2), (−1,4) and (−2,−10)—these are shares. To recover the secret, one need to reconstruct the polynomial from any three points (which is possible even if one of four points is missing) and get free term. Obviously polynomial formula is shredded after share generation and cannot be used to read the secret directly.

Many more secret sharing algorithms are available besides presented above [17,18,19,20,21,22,23,24]. They offer various secret formats, number of shares and other characteristics that may be adjusted to needs.

## 5. Distributed Steganography on Hidden Pages in PDF

Previous sections explained how to conceal a page in PDF file and how to divide a secret into pieces. In distributed steganography both these operations are used. Each share is embedded in separate document by placing the payload at additional page which is later removed (as presented in Section 3.1). In reconstruction phase, shares are combined to form the secret. But in between there is one more thing to discuss: hidden data recovery.

To explain that, a few details of PDF processing will be necessary. The data in PDF files are stored in binary streams that are easy to identify: they lie between stream and endstream keywords. The content of the stream may be additionally encoded (which is optional, but very often used due to compression) or encrypted. An object with stream may declare many encodings which are applied subsequently. In such case, to retrieve final data, multiple decoders need to be used one by one.

When the document is processed, the software is able to identify required decoders because of the property /Filter followed by decoder name. The most common is /FlateDecode (zlib compression), but others like /ASCIIHexDecode are also possible.

### 5.1. Example of Decoding a Secret from Multiple PDF Files

To show presented ideas in practice, we will go step by step through decoding process. The message is split with XOR technique (described in Section 4) and hidden in two documents filled with “Lorem ipsum” text (2 and 5 pages). First we need to locate the removed page and follow the references until reaching the object with binary stream. In first file its content was (non-printing characters are in hex):


x\x9c=\xcb\xb1\n\xc30\x0c\x04\xd0]_\xa1\xd1\x81\xda\x91,\xdb\xb2\xd6@\x96n\x05o\xa5C) $S\x86\xa6\xff\x0f5))\x82\xe3\x1d\x87\x18\xa9\x9f\xe7\x1e5q\xa8\xd5\xb4$|m\xf0\x06: \xa6}\xc5\xf1I\xb8~‘j‘\xc1J\xcc&\xbf\xa7\x7fc\xb1\xa0)\x1b\xa5\xce\x1c\xb40i\xc5\xb6 \xc1\xb8x \xf6\x84\x8cm\x81\xbbc\xbe\x0c^$\xba(vH\x1c\xab\x9e\xe2SQ\xca\xf0hW\x98 \x1b\xdc\xe0\x0b#\xec \x1f


The object declared FlateDecode, therefore the next step is to decompress these data with zlib. At this stage the interesting parts are already visible:


1 0 0 -1 0 841.889764 cm
q
0 0 0 rg /a0 gs
BT
9.962593 0 0 -9.962593 139.745904 135.761078 Tm
/f-1-0 1 Tf
[(11,)-332(239,)-333(177,)-333(117,)-333(236)]TJ
ET
Q


From that we can easily obtain pure numbers: 11, 239, 177, 117, 236.

The same process we may repeated for second file. Here is binary content of the stream:


x\x9c=\xcb;\x0b\xc30\x0c\x04\xe0]\xbfBc\x02\xb5\xa3\xb3\xfc\xd2Z\xe8\xd2\xad\xe0\xad t(\x85d\xca\xd0\xf4\xffCM\xfa@p|\xc7!\xb0\xf4 s\xe8Q#|\xadVr\xe4\xc7JO\x92}\xda\x16 \x9e\xee\xc2\xcb\x8b\x8e\x8d\xcc[\x0e\xc9\xf4\xf3\xf4oP\xf3%&\x93\xd8\x99|\xc9\x90R \xb9\xad4 \xcd\x0eN\x18\xdcf\xba\x0e\xb9\x1cF\xa7\x1a\x06h\xdd\xa5C\x08\xf8)}\x01\xc5 xkg:5\xba\xd0\x1b\x04[\x1f\xe6

After zlib decompression:


1 0 0 -1 0 841.889764 cm
q
0 0 0 rg /a0 gs
BT
9.962593 0 0 -9.962593 139.745904 135.761078 Tm
/f-1-0 1 Tf
[(67,)-332(138,)-333(221,)-333(25,)-333(131)]TJ
ET
Q


And just numbers: 67, 138, 221, 25, 131.

The one final step is needed to decode hidden message. Two arrays should be XORed elementwise and casted to characters. Eventually the secret is revealed: *Hello*.

## 6. Detection

It is also important to analyze possible attacks aimed at detecting or destroying hidden data. When the original file is present, an adversary may use diff program to find discrepancies. However, PDF documents contain binary streams, so they are automatically treated as binaries. Normally, when diff compares binaries, it only informs whether the files differ or not, without giving any details or showing interesting fragments. This is why the adversary needs to use --text option to compare PDF documents line by line.

**Figure 5:**
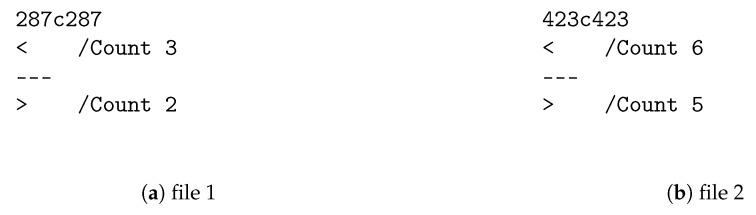
Output of diff program for modified files in ideal case.

The program deals well with this task, especially in ideal case when only the counter was modified (Figure 5). These are files that were analyzed in Section 5.1. The output shows how exactly they differ. For example, “287c287” means that line 287 from first file need to be changed (c) to match line 287 from second file. The lines themselves are separated with “---” and each of them is marked with “<” or “>” depending of origin.

Such laboratory conditions obviously do not apply to real life in which source file is not conveniently available. Then the adversary needs to perform deeper analysis, including tree structure examination. One possibility is to look for orphaned objects (those which cannot be reached from the root). This strategy is eventually able to find objects deleted from Kids references. On the other hand, it is useless when only the counter was decreased and the references are present. So the attacker should also check pages table and counter value. Different approaches, like manual examination of objects, are more probable to fail. In PDF documents numerous objects represent fonts and page elements, so finding actual content may be challenging.

Incidentally, detecting additional objects or other errors in PDF file does not necessarily mean that steganography was used. Sometimes documents are malformed, have some parts missing or present other weirdness. But the software is still able to process those files and display them more or less correctly. The question may arise why invalid documents are accepted, usually without any warning? The answer is simple: if the reader forced precise adjustment to standard, some documents would break and users would switch to another program that supports not-so-correct format (speaking of which, the same problem is present in HTML browsers). A positive side of that mess is the chance to occasionally use steganography in PDF documents.

Another method of steganalysis is file size examination. This technique is efficient in very short files or when payload size is significant in relation to carrier document size. Of course in serious applications such situation is unacceptable—when size of the file changes noticeably, another container should be selected. Nevertheless, the method is mentioned as theoretical mean which may be used against inexperienced steganogaphers.

Presented technique is resistant to attacks targeted at file content (like resampling and replacing an image) because it is structure-based. The resistance to joining PDF documents varies depending on the software used. It was tested with two applications: pdftk and pdfmerge. The interesting thing is that pdftk behaves differently when working on files created with various version of the algorithm. In case of simple counter decrement it leaks share content, but when the references are deleted, the shares are not compromised (they may be removed, though). These observations apply regardless of order of concatenated documents. On the other hand, pdfmerge raises an error when at least one of the input files is modified, but nonetheless creates valid output without revealing hidden shares.

Destroying hidden data is separate type of attack that is aimed to remove the payload, not to detect it. In typical blind usage suspicious file is processed regardless whether it contains secret data or not. This approach may be used when the amount of data is so massive that it exceeds the ability of inspection or when other methods fail. Such attacks give quite good results in image steganography, because human eye is unable to notice slight alterations of pixels. However, the same cannot be said about binary data. Blind modification or deletion of random streams may damage the file, change its appearance or destroy important content. In conclusion, blind attacks to PDF documents usually make those files useless—for this reason are not recommended.

## 7. Summary

The concept presented in this paper has been shown on some short documents to be easy to understand. But the analysis reached also larger PDF files, including over 300 MB file created from scanned images. Regardless of page dimensions or dominating types of objects, the hiding and recovering processes were successful. This means that different kinds of PDF documents may serve as containers (scanned files, presentations, text documents, etc.), which is a huge advantage of presented method. Also the secret length may vary. The tests proved that it is possible to conceal a secret on single page as well as on multiple pages. So the universality is main factor that distinguishes this technique from others and allows to build upon it for further advancements.

The solution was analyzed with 7 different programs: 4 PDF readers (Evince, Adobe Acrobat X, Okular, Firefox—web browser) and 3 graphical applications with ability to render PDF content (GIMP, Inkscape and Draw from LibreOffice). Created files opened correctly in all of them; the approach of decrement the counter turned out to be better, because the software had not shown any trace of hidden content.

In real world applications the circumstances should also be considered to make detection harder. The recommended way is to use common documents of decent length that may easily blend in normal traffic. Although the capacity of presented method is theoretically unlimited, it is advisable to choose carriers so that the share size does not exceed 1% (cautious variant) to 5% (more risky) of carrier size. It is particularly important when the shares are not textual, but for example in graphical format.

PDF files have been chosen to this study because of their wide use in Internet which make them great steganographic medium. Obtained results encourage to conduct more research in this area. The future projects may concern new methods of distributed steganography in PDF documents, incorporating cryptography and other means of security. Another idea is to extend the study by new types of carriers and develop advanced methods of sharing and concealing secrets. This covers a broad area of research and concerns especially combination of steganography and secret sharing [25,26]. There are still much things to discover and open problems to address in fascinating field of information hiding.

## Abbreviations

The following abbreviations are used in this manuscript:

PDF	Portable Document Format
ASCII	American Standard Code for Information Interchange
HTML	Hypertext Markup Language
